# Utilising the perspectives of patients with lower-limb osteoarthritis on prescribed physical activity to develop a theoretically informed physiotherapy intervention

**DOI:** 10.1186/s12891-021-04036-8

**Published:** 2021-02-08

**Authors:** Matthew Willett, Carolyn Greig, Sally Fenton, David Rogers, Joan Duda, Alison Rushton

**Affiliations:** 1 Centre of Precision Rehabilitation for Spinal Pain, University of Birmingham, Birmingham, UK; 2 School of Sport, Exercise and Rehabilitation Sciences, University of Birmingham, Birmingham, UK; 3 MRC-Versus Arthritis Centre for Musculoskeletal Ageing Research, University of Birmingham, Birmingham, UK; 4 NIHR Birmingham Biomedical Research Centre, University Hospitals Birmingham NHS Foundation Trust and University of Birmingham, Birmingham, UK; 5 Centre for Musculoskeletal Medicine, Royal Orthopaedic Hospital NHS Foundation Trust, Birmingham, UK; 6 School of Physical Therapy, Western University, Elborn College, London, Canada

**Keywords:** Theoretical intervention, Osteoarthritis, Physiotherapy, Behaviour change

## Abstract

**Background:**

Lower-limb osteoarthritis (OA) causes high levels of pain and disability. Physiotherapists are the primary healthcare provider of non-pharmacological treatments, and incorporate strategies to optimise physical activity (PA) to aid patients with lower-limb OA to moderate their clinical symptoms. However, patients with lower-limb OA have low adherence to PA recommendations both during treatment and after discharge. This study aimed to use knowledge of identified barriers and facilitators to physiotherapy prescribed PA (during treatment and post-discharge) to develop a theoretically informed intervention to optimise adherence to PA for patients with lower-limb OA during treatment and post-discharge.

**Methods:**

1) A purposive sample of 13 patients with lower-limb OA participated in semi-structured interviews following physiotherapy treatment. Inductive analysis identified themes/subthemes reflecting barriers and facilitators to physiotherapist prescribed PA, which were organised deductively according to personal factors, treatment and post-discharge phases.

2) Themes/subthemes were mapped onto the *theoretical domains framework* (TDF).

3) Behaviour change techniques (BCTs) were coded from the key identified domains and a theoretically informed physiotherapy intervention addressing barriers and using facilitators, was developed.

**Results:**

Themes of patient confidence, mind-set, motivation, OA symptoms and PA experiences were primary personal factors that influenced PA adherence; with the TDF domain *‘Beliefs about capabilities’* most important to target. During treatment, the theme of routine formation was the major driver of personal factors; and primarily influenced by developing a positive physiotherapist-patient relationship. Post-discharge, physical factors, psychosocial factors and ongoing access to resources were important themes influencing PA maintenance. *‘Environmental context and resources’ and ‘social influences’* emerged as the key TDF domains to target during treatment and post-discharge. The proposed theoretically informed intervention included 26 BCTs delivered across conceptual phases of adoption, routine formation, and maintenance.

**Conclusion:**

A theoretically informed physiotherapy intervention was proposed to optimise PA adherence in patients with lower-limb OA. The included BCTs primarily target patients’ perceived beliefs about their capabilities, by developing a PA routine during treatment and facilitating appropriate psychosocial support and access to resources for PA maintenance post-discharge. The feasibility of delivering the intervention in clinical practice will now be evaluated.

**Supplementary Information:**

The online version contains supplementary material available at 10.1186/s12891-021-04036-8.

## Background

Osteoarthritis is a leading cause of pain and reduced function and quality of life [1]. In the United Kingdom (UK), OA is a burden on health services and the greatest cause of individual level disability in people aged 45 years or older, with approximately 2 million general practitioner (GP) visits each year related to OA symptoms [2]. The large synovial joints of the lower-limb (hip and knee) are the most common cause of OA related pain, accounting for approximately 70% of symptoms [3]. As there is currently no cure for OA [2], and with increased life expectancy [4], it is likely that greater numbers of patients with lower-limb OA will be required to self-manage their symptoms to reduce the load on healthcare systems in the coming years.

Promoting physical activity is a key non-pharmacological strategy that healthcare guidelines recommend to aid patients with lower-limb OA to manage their symptoms [1, 2, 5, 6]. However, the majority of patients with lower-limb OA are less active than asymptomatic populations [7], and healthcare PA interventions are generally only effective at reducing short-term (≤ 3 months post baseline) [8] symptoms, with pain and loss of function returning after about 6 months [9]. This is likely associated with a gradual reduction in patient adherence to prescribed PA after discharge, when approximately 90% of patients with lower-limb OA do not maintain their PA [10].

People undergo several ‘phases’ of behaviour change when integrating new behaviours into their lifestyle [11]. The most important phases are *‘adoption’* [12, 13], which occurs while people are receiving treatment from a health professional, and *‘maintenance*’ [14] which would be ongoing post-discharge and occurs after 6 months of behavioural practice. While adoption and maintenance have several overlapping influences, they also possess unique determinants [11, 15, 16]. Therefore, a healthcare intervention needs to incorporate specific behaviour change techniques (BCTs) to match these phases in the behaviour change process to optimise PA adherence.

Physiotherapists are the primary healthcare provider of non-pharmacological treatments for patients with lower-limb OA [17]. As such, they are in an optimal position to promote PA adherence [18]. However, physiotherapists and patients with lower-limb OA do not necessarily agree with the most effective BCTs to promote PA adherence [19]. Furthermore, patients with lower-limb OA believe they require more support and can perceive prescribed PA (e.g. exercise) as unsafe [20]. Physiotherapists also perceive they do not have sufficient understanding of BCTs to deliver them consistently in practice [21].

The Medical Research Council (MRC) advocates that interventions should be informed by behaviour change theory [22] to enable a greater understanding of the assumed intervention mediators (e.g. self-efficacy) effects on the target behaviour to enable refinement for future testing, and therefore, greater clinical effectiveness over time [23]. The Theoretical Domains Framework (TDF) is a validated framework which synthesises constructs from 33 theories of behaviour change into 14 overarching domains [24, 25]. By mapping barriers and facilitators to specific domains, the TDF can be used to identify key determinants of behaviour change [26] and important BCTs to develop theoretically informed interventions [27, 28].

Qualitative methods are utilised to provide an in-depth understanding of patients experiences and identify barriers and facilitators to treatment [29]. Although previous qualitative studies [30–38] have determined barriers and facilitators to PA for patients with lower-limb OA, only one study has considered stages of behaviour change in their analysis [39]. Hammer et al. [39] found that patient self-efficacy and severity of symptoms were highly influential on PA maintenance. However due to their long-term focus, it was problematic to identify similarities or differences in determinants that occurred while under treatment (related to adoption) or post-discharge (related to maintenance) and their results were not utilised to develop a behaviour change intervention. Currently there are no theoretically informed interventions that incorporate BCTs that target the overlapping and unique features of PA adherence during the adoption *and* maintenance phases. Therefore, this study aimed to gain an in-depth understanding of the overlapping and unique barriers and facilitators to physiotherapist prescribed PA that patients with lower-limb OA experience during treatment and post-discharge to develop a theoretically informed physiotherapy intervention.

### Objectives

1. To explore patients’ in-depth experiences of barriers and facilitators to physiotherapist prescribed PA within sessions that effect adherence while receiving treatment and post-discharge.
2. To use the barriers and facilitators to identify themes/subthemes that influence patient’s adherence to prescribed PA during treatment and post-discharge.
3. To map themes/subthemes to the TDF to identify key domains that effect adherence to PA during treatment and post-discharge.
4. To identify appropriate BCTs from the TDF domains to propose a theoretically informed intervention aimed at optimising PA adherence.

## Methods

### Theoretical framework

**Fig. 1:**
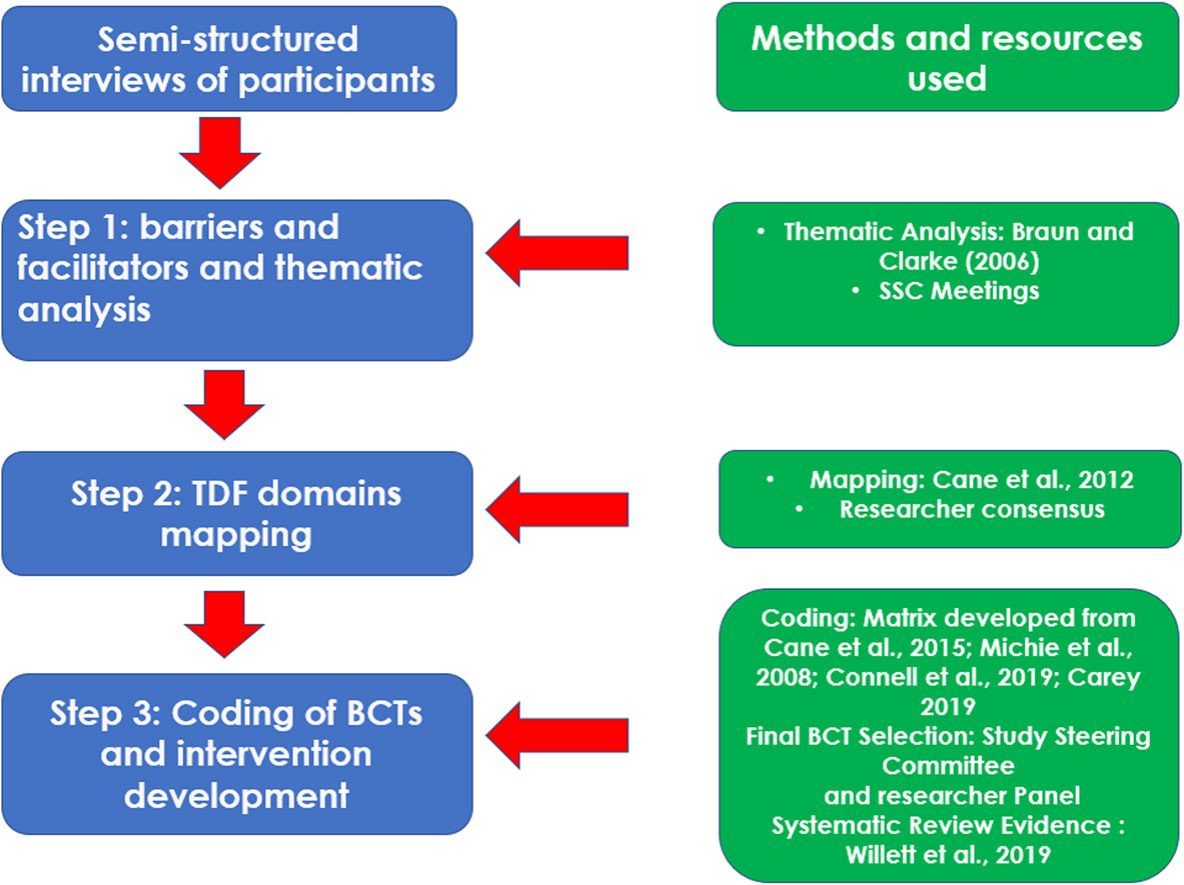
Overview of intervention development process. Legend: TDF: Theoretical Domains Framework; BCT: Behaviour Change Technique

The in-depth perspectives of patients with lower-limb OA and their individual experiences of attending physiotherapy for the management of their OA related symptoms were sought to propose a theoretically informed intervention [40]. This study utilised a phenomenological framework [41], which followed a published protocol [42], and was reported using the COnsolidated criteria for REporting Qualitative research (COREQ) [43]. A summary of the study processes is outlined in Fig. 1.

### Research team and reflexivity

One-to-one semi structured interviews were conducted by the lead researcher (MW); an experienced physiotherapist who has treated patients with lower-limb OA for > 15 years. Participants were informed of MW’s occupation, but his role as a researcher was emphasised. Participants had not met MW prior to the interview.

### Participant selection

Adult patients (≥18 years) with hip or knee OA (diagnosed through NICE guidelines [2] or imaging), who had attended physiotherapy at the Royal Orthopaedic Hospital (ROH), in Birmingham, England, were recruited. The target sample comprised those who had experienced the phenomenon of interest (i.e. patients with lower-limb OA who had received physiotherapy treatment and would likely have perceptions of the barriers and facilitators to prescribed PA) and therefore, purposive sampling was utilised [44]. Initially, stratified purposive sampling [45] aimed to recruit approximately 60% of participants with knee OA and 60% being female [46] (based on UK epidemiological data). Theoretical purposeful sampling [45] was then utilised in the final stages of recruitment to ensure secondary demographic factors (e.g. working status, age, educational background) that may influence patient perspectives could be included in the analysis [47]. Sampling continued until data saturation (the point where no new themes emerge from the data analysis) had been achieved [48]. Within phenomenology, a sample of up to 10 is generally accepted to reach data saturation [44]. However, Francis et al. [48] advocated that a further three interviews should be conducted post saturation, to ensure no new themes emerge. Therefore, a sample of 10–15 participants was anticipated which was consistent with previous phenomenological studies on patients with lower-limb OA [30, 31, 39].

Potential participants were identified by their treating physiotherapist and the study was outlined at the final treatment session. Each interested patient was given a participant information sheet (PIS) and asked to sign a consent to contact form passed to the site lead investigator (DR) who sent the form to MW by encrypted email. MW telephoned potential participants to verify eligibility, outline the study, answer any queries, and negotiate a time and place for the interview to take place. Potential participants were given at least 1 week to enable them to make an informed decision regarding participation. Participants could have interviews at their home, the ROH or at the University of Birmingham.

### Data collection

The interview schedule (Additional file 1) was developed by integrating content from contemporary research examining the perspectives on PA in patients with lower-limb OA [30, 34, 35, 49] and refined by piloting with the study’s patient and public representation (ED) who has lower-limb OA. The schedule included questions on patients’ current levels of PA (adapted 3 day Physical Activity Recall interview [50, 51]) and key demographic data (gender, age, Body Mass Index (BMI)), ethnicity, length of time since OA diagnosis and their highest educational level. Several patient reported outcome measures were integrated into the interview schedule: bothersomeness [52] and average pain [53] of their symptoms over the previous week, and either the short form Hip [54] or Knee [55] Injury and Osteoarthritis Outcome Scores (HOOS/KOOS). Open questions (with prompting where required) were used to gain participants’ in-depth opinions on: the received physiotherapy intervention and key barriers and facilitators or BCTs that influenced their prescribed PA during treatment and post-discharge. Participants were invited to offer any further perspectives that they felt were not addressed in the topic guides. Interviews were audio-recorded, transcribed verbatim, and mailed/emailed (dependent on preference) to participants for member checking so researcher interpretation of the data could be reviewed [56]. No follow up interviews were planned.

### Data analysis and development of intervention

Development of the theoretically informed physiotherapy intervention followed a modified version of the steps outlined by French et al. [57].

#### Step 1: identification of barriers and facilitators and thematic analysis

Barriers and facilitators to physiotherapists prescribed PA were identified and synthesised to generate themes and subthemes following thematic analysis guidance as outlined by Braun and Clarke [58] (Objectives 1 and 2).

##### Familiarisation with the data and generating initial codes

MW took field notes to summarise key thoughts at salient points of the semi-structured interviews which enabled initial familiarisation with the data. Transcribed verbatim interviews were uploaded into NVivo software and read several times by two researchers (MW, SF) who attempted to put aside their pre-conceived thoughts to maintain the unique experiences of the participants in the analysis [44]. MW and SF coded the first transcript in tandem and the second independently to establish consistency [59]. The researchers included additional text so that each code maintained its own meaning [44], with as many examples as possible included to ensure contextual factors were recorded [58]. Any explicit details outlining participants perspectives on specific BCTs and/or their mode of delivery or perspectives on sessions (i.e. number, frequency or duration) that appeared in the transcript were noted by the coders to ensure that the proposed intervention matched participants suggestions. MW and SF worked collaboratively to arrange codes (with quotations) from the first transcript into preliminary themes/sub-themes with several follow up meetings used to iteratively update the emerging results from the second transcript. The remaining transcripts were coded by MW.

##### Searching for themes

The interview topic guide included questions outlining barriers and facilitators during treatment and post discharge, and those which were intrinsic to the participants experience. Therefore, a deductive framework was utilised initially to cluster preliminary codes and quotations (including both barriers and facilitators) into three overlapping groupings of themes.

1. ‘Personal factors’ grouping encompasses themes which could influence PA adherence across both during treatment and post-discharge.
2. ‘Treatment factors’ grouping encompasses themes that primarily influenced PA adoption including effects that the physiotherapist and treatment sessions had on patients.
3. ‘Post-discharge factors’ grouping encompasses themes that primarily influenced PA maintenance beyond the clinic after the patient is discharged from care.

##### Reviewing, defining and naming themes

Data on NVIVO were reviewed by MW who grouped barriers and facilitators to develop preliminary themes and subthemes. MW wrote all subthemes and supporting quotations on post-it notes and generated an initial inductive mind-map which enabled visual exploration of patterns of themes and subthemes including any overlap and delineation of individual perspectives and experiences [60]. Common subthemes and deviant cases were sought to ensure individual perspectives were captured within the analysis [44]. The mind-maps were videoed with theme associations outlined for later review. Emerging themes/subthemes and mind maps were audited and clarified through presentations to the chief investigator (AR) and results, supported by quotations, were developed iteratively through presentations and feedback at Study Steering Committee (SSC) meetings. The SSC included researchers, clinicians, and patient and public involvement and the in-depth, collaborative feedback aided the analyses’ value and the studies reflexivity [61].

#### Step 2: mapping to the theoretical domains framework

To provide a comprehensive overview of associated domains, all subthemes were mapped onto the TDF (objective 3). TDF Mapping was piloted by two researchers (MW, SF) over a series of meetings and supported by a modified manual from a previous study [62]. Mapping was completed by MW and audited by SF with any issues discussed. In the case of any mapping disagreements, an expert in behaviour change theory (JD) mediated. If agreement of mapping a subtheme to single domain could not be reached, it was mapped onto all domains identified by either coder [26]. Each subtheme was evaluated across three criteria to clarify its relative importance [63]: The number of participants who identified each subtheme; the presence of strong beliefs; the presence of conflicting beliefs. The domains outlined from the subtheme mapping were synthesised to identify the most important domains for each theme for the intervention to target.

#### Step 3: behaviour change technique identification and intervention development

A coding matrix (Additional file 2), synthesising research linking BCTs to theoretical constructs [27, 28] and mechanisms of action [64, 65] was used to identify all potential BCTs from the key TDF domains within each theme (objective 4). BCTs were categorised as ‘unique’ if they were coded exclusively/primarily to a one grouping, or ‘overlapping’ if they were identified consistently across groupings (Additonal file 2). To reflect clinical practice and enable capture of the phases of behaviour change, the proposed intervention was split into early (adoption), middle, and late (maintenance) sessions. BCTs coded to the treatment or post-discharge grouping were incorporated into the early-middle sessions to target PA adoption, or middle-late sessions to target PA maintenance respectively. Those that were coded to the personal factors grouping, or across groupings, were generally considered reoccurring and repeated across sessions. Although the aforementioned studies [27, 28, 65] offer a useful way to identify BCTs, there is currently no guidance regarding prioritisation or mode of delivery [66]. Therefore, if conflicts were identified during BCT identification, Cane et al. [28] was preferentially used as it is the only study that has demonstrated reliability and validity when linking BCTs to behavioural domains. The final selection of BCTs, including their mode of delivery and physiotherapy session detail (i.e. suggested number of sessions, duration, and frequency), was further informed by: repeated review of participants transcripts; collaborative discussions at SSC meetings; the lead investigators clinical experience; the BCTs most commonly used in clinical practice [28]; the most effective BCTs from low risk of bias trials identified in by our systematic review [9].

## Results

### Participants

All 13 patients (*n* = 4 males) who were approached agreed to participate and interviews were conducted March–November 2019 and lasted 46–70 min (mean 56 min). All participants were of White British descent, ninewith knee OA, three with unilateral hip OA, and one with severe hip and mild bilateral knee OA (considered as a hip participant in the analysis). Although two participants had family members in the house during their interview, no person other than the participant and interviewer were present in the interview room. Saturation of themes was reached after 10 participants, and a further three interviews ensured no further themes emerged [48]. Participants’ ages ranged between 44 and 76 years (mean 63; standard deviation 10.7) and BMI ranged between 24 and 42 (31.9; 5.3). Participants’ average pain intensities ranged from 1 to 6 (3.9;1.9) and six participants rated their bothersomeness as ‘moderately’ over the past week. The SF-KOOS and HOOS scores ranged from 24.9–66.6 (38.77;11.35) and 33.9–46.1 (40.9; 4.4) respectively. PA levels ranged from 1975 to 4250 kcal/day for the previous 3 days with a mean of 2920.8 (660.0) kcal/day. Twelve participants had their diagnosis confirmed with an X-ray and one with patient history and clinical examination data. Symptom duration varied from < 1 year to 48 years. Participant data are shown in Table 1. Transcripts were sent to six participants at their request, but none responded with comments.

**Table 1:** Participant demographic features

Participant no	Gender	Age	BMI	Joint	NPRS	BSM	SF KOOS	SF HOOS	PA	HEL	TSSB	TSD	EM
1	F	60	26	Right knee	1	Slightly	35.3	N/A	2155	O levels	2	0.5	N
2	F	75	35	Right knee	2	Moderately	46.1	N/A	2588	PG Diploma	25	25	N
3	M	75	33	Both knees	6	Very much	42	N/A	3800	High School	30	0.75	N
4	F	63	28	Right knee	3	Moderately	33.6	N/A	2692	UG degree	1.5	0.5	Y
5	M	76	25	Left knee	5	Slightly	35.3	N/A	2580	Left school at 14	48	3	N
6	F	53	42	Right knee	6	Very much	66.6	N/A	4250	Left School at 16	12	1	Y
7	F	75	35	Right hip; both knees	4	Moderately	37	41.7	2672	College	6	4	N
8	F	63	24	Left knee	6	Moderately	42	N/A	2376	UG degree	4	1	Y
9	F	67	37	Left hip	3	Slightly	N/A	33.9	3197	UG degree	0.5.	0.5	Y
10	F	53	27	Left knee	2	Slightly	24.9	N/A	1975	College	6	5	
11	F	67	N/G	Both knees	1	Slightly	24.9	N/A	N/G	High School	10	10	Y
12	M	44	30	Left hop	6	Moderately	N/A	41.7	3201	College	2.5	0.5	Y
13	M	48	29	Right hip	6	Moderately	N/A	46.1	3564	Master’s Degree	6	3	Y

*no* Number, *BMI* Body Mass Index, *NPRS* Numerical Pain Rating Scale, *BSM* Bothersomeness, *SF KOOS* Short Form Knee Injury and Osteoarthritis Outcome Score, *SF HOOS* Short Form Hip Injury and Osteoarthritis Outcome Score, *PA* Physical Activity level (mean kcal/day for past 3 days), *HEL* Highest Education Level Achieved at time of interview, *TSSB* Time since symptoms began, *TSD* Time Since diagnosis of OA, *EM* Employed at time of interview

#### Step 1: identification of barriers and facilitators and thematic analysis and step 2: mapping to the theoretical domains framework

Due to the integrative nature of the data analysis, and to avoid repetition, the results for Steps 1 and 2 are presented together. A summary of themes within each deductive grouping, and their associated primary TDF domains are presented in Table 2. Overall, 13 of 14 TDF domains (all accept ‘*attention and decision-making processes*’) were identified as important influences of PA adherence in at least one theme. Nine TDF domains were identified within the personal factors grouping with ‘*Beliefs about Capabilities*’ influential to all themes. The treatment phase grouping identified ten TDF domains as important with ‘*Environmental Context and Resources’* and ‘*Social Influences’* associated across three themes. Seven TDF domains were identified in the post-discharge grouping with domain ‘*Environmental Context and Resources’* important across all themes. Additional detail on subthemes and TDF mapping, with example supporting quotations are included for the personal factors, treatment and post-discharge groupings in Additional file 3: Tables 1-3 respectively and further detailed below.

**Table 2:** Key theoretical domains frameworks domains that influenced themes

Grouping	Theme	TDF Domain													
		Kn	Sk	SPRI	BACap	Op	BACon	Rein	Intent	Goals	MAD	ECR	SocI	Em	BehR
Personal factors	Motivation				✓				✓	✓				✓	
	Confidence	✓	✓		✓		✓								
	Mindset				✓	✓						✓		✓	
	Pain	✓			✓	✓								✓	
	Previous Experience		✓		✓		✓	✓							
Treatment Phase	Routine	✓	✓					✓	✓	✓		✓			✓
	Access											✓			
	Value											✓	✓		
	Relationship					✓							✓		
	Personalised Rx	✓	✓		✓			✓	✓	✓			✓		✓
Post-Discharge Phase	Access							✓				✓	✓		
	Psychosocial factors			✓	✓	✓						✓	✓		
	Physical factors				✓		✓					✓			

*TDF* Theoretical Domains Framework, *Kn* Knowledge, *Sk* Skills, *SPRI* Social/Professional Role and Identity, *BACap* Beliefs about Capabilities, *Op* Optimism, *BACon* Beliefs about Consequences, *Rein* Reinforcement, *Intent* Intention, *MAD* Memory, Attention and Decision Processes, *ECR* Environmental Context and Resources, *SocI* Social Influences, *Em* Emotion, *BehR* Behavioural Regulation

#### Personal factors grouping

Five interrelated themes contributed to the personal factors grouping; participant’s motivation, confidence, mindset, arthritic symptoms, and experiences of PA (Additional file 3: Table 1).

##### Motivation theme

Patients motivation to adhere to PA was influenced by a continuum of factors and included internal and external sources. Subthemes related to internal sources of motivation included: enjoyment (or not) of PA; seeing progress; having a purpose; wanting to maintain function or moderate arthritic symptoms; fear of functional decline or need of invasive surgery; and a sense of personal-responsibility or guilt at not doing prescribed PA. Subthemes related to external sources of motivation were; use of the physiotherapist or reminders (e.g., use one’s Theraband) as motivators. The most important TDF domains associated with motivation were ‘*Intentions*’, ‘*Goals*’, and ‘*Emotion*’, and ‘*Beliefs about Capabilities’*.

##### Confidence theme

Participant confidence was highly influential in promoting PA adherence. Subthemes included: the effect of PA on symptoms and function; feelings of PA capability; and fear avoidance. The most important TDF domains associated with this theme were ‘*Knowledge*’, ‘*Skills*’, ‘*Beliefs about Capabilities*’, and ‘*Beliefs about Consequences*.’

##### Mindset theme

Several participants outlined the importance of a positive mindset, or having the mental energy or headspace to engage with prescribed PA. This theme was very closely linked with participant confidence to undertake PA and included the subthemes: psychological effects of symptoms and PA; mental and emotional resources; and comfort zone. The most important TDF domains were ‘*Beliefs about Capabilities’*, ‘*Optimism*’, ‘*Environmental Context and Resources*’, and ‘*Emotions*’.

##### Arthritic symptoms

Pain and other arthritic symptoms (e.g. articular stiffness) were key influences of PA adherence. Subthemes included the effect of symptoms on: Undertaking PA; mood; enjoyment of PA; and the effect of pharmacology on pain. The key associated TDF domains were ‘*Beliefs about Capabilities*’, ‘*Knowledge*’, ‘*Optimism*’, and ‘*Emotion*’.

##### Experiences of PA

Participants’ previous PA experiences influenced their PA adherence. Sub-themes included: PA effects on participants wellbeing, mind-set, arthritic symptoms and function; and participants identifying oneself as physically active; their expectations of treatment; and frustration at loss of function. The most important associated TDF domains were ‘*Skills*’, ‘*Beliefs about capabilities*’, ‘Beliefs about Consequences’, and ‘*Reinforcement*’.

#### Treatment phase factors grouping

The treatment phase grouping included the themes: routine formation; person-centred treatment; the patient-physiotherapist relationship; access; and value of physiotherapy sessions (Additional file 3: Table 2).

##### Routine formation

PA routine formation emerged as the primary mechanism to promote patient confidence, motivation, and a positive mindset. Subthemes were grouped into barriers and facilitators. Facilitators included: having a PA plan; integrating PA into daily life; promoting habits; and the effects of routine on motivation and well-being. Barriers to routine included: health concerns; memory; other commitments; and time. The primary associated TDF domains were ‘*Knowledge*’, ‘*Skills*’, ‘*Intentions*’, ‘*Goals*’, and ‘*Behavioural Regulation’* which contained both barriers and facilitators and ‘*Environmental Context and Resources’* which was exclusively identified as a barrier to this theme.

##### Person-centred treatment

A collaborative, person-centred treatment approach was important to developing a PA routine. Subthemes included: a personalised treatment plan; a detailed PA routine; ongoing assessment and reassessment; and understanding of OA. The primary associated TDF domains were ‘*Knowledge*’, ‘*Skills*’, ‘*Reinforcement*’, ‘*Intention*’, ‘*Goals*’, and ‘*Behavioural Regulation’*.

##### Relationship with the physiotherapist

Participants outlined the importance of their relationship with the physiotherapist in adhering to prescribed PA. Subthemes included: personal level knowledge; collaborative relationship; familiarity; encouragement (from physiotherapist); motivation (from physiotherapist); communication; and the physiotherapist’s attitude. The most important TDF domains were ‘*Optimism*’ and ‘*Social Influences’*.

##### Access to physiotherapy sessions

Participant’s access to their physiotherapist influenced the development of a PA routine. Subthemes included: other commitments; appointment flexibility; number and of sessions; and referral process. The primary associated TDF domain theme was *‘Environmental Context and Resources’.*

##### Value of physiotherapy sessions

PA adherence was influenced by how valuable participants perceived their physiotherapy sessions to be. Subthemes include: motivation from sessions; empowerment from sessions; too much talking; and PA within sessions. The primary associated TDF domains were *‘Environmental Context and Resources’* and *‘Social Influences’.*

#### Post-discharge factors grouping

The post-discharge grouping included the themes: access to resources; psychosocial factors; and physical factors (Additional file 3: Table 3).

##### Access to resources

Access to a community facility or physical space influenced post-discharge PA maintenance. Subthemes included: Follow-up with the physiotherapist; Appropriate facilities and; Ease of access. The primary associated TDF domains were ‘*Environmental Context and Resources’* and ‘*Social Influences*.’

##### Psychosocial factors

Ongoing psychosocial support influenced PA post-discharge. Subthemes included: Relatedness to others; Fear of judgement; and Psychosocial support. The most important TDF domains were ‘*Environmental Contexts and Resources’*, ‘*Social Influences’*, ‘*Social Identity’*, ‘*Beliefs about Capabilities*’, and ‘*Optimism*’.

##### Physical factors

Physical factors impacted participants adherence to PA post-discharge. Subthemes included: Perceived physical capability; intrinsic physical factors (e.g. age, weight); and extrinsic physical factors (e.g. hills, weather). The primary associated TDF domains were ‘*Beliefs about Capabilities*’, ‘*Beliefs about Consequences*’ and ‘*Environmental Context and Resources’*.

#### Interrelationship of themes across groupings

**Fig. 2:**
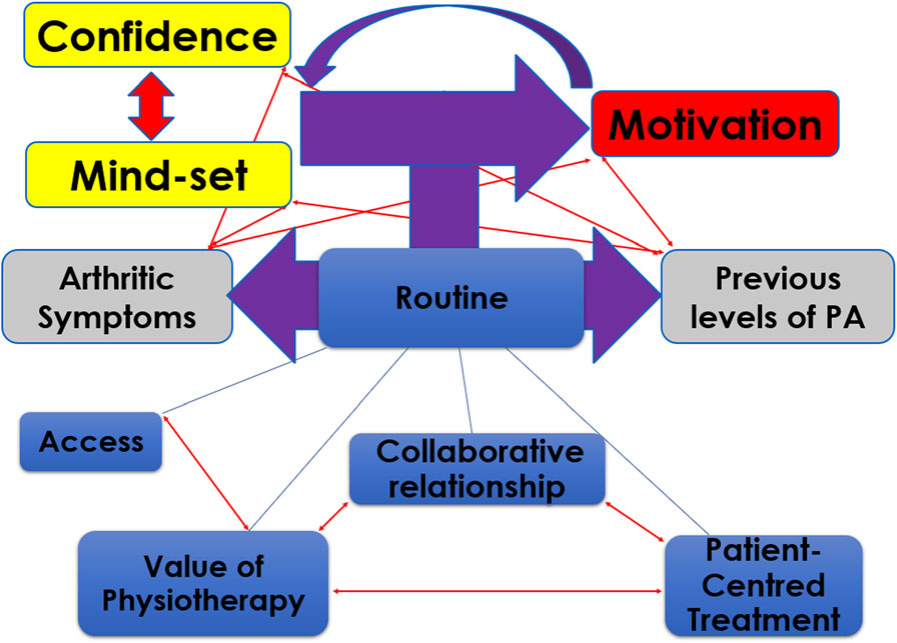
Conceptual mindmap of Personal and Treatment Themes influencing Adherence to Physical Activity

**Fig. 3:**
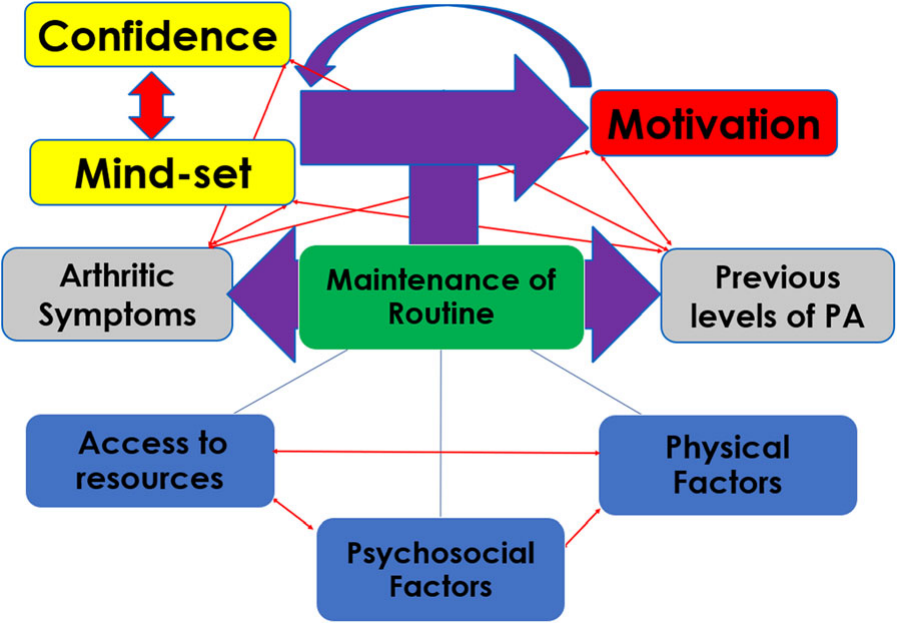
Conceptual mindmap of Personal and Post-Discharge Themes influencing Adherence to Physical Activity

The relationships of the themes within the personal factors and treatment and post-discharge groupings can be visualised in Figs. 2 and 3 respectively. The conceptual mind-maps demonstrate the importance of the participant’s mindset and confidence to adhere with prescribed PA and their influence on motivation. The smaller curved arrow details the feedback and ongoing relationship between these personal factors. The figure also takes into account the interrelation of patients unique PA experiences and OA symptoms with the other personal factors. The main vertical arrows demonstrate PA routine as the key mechanism in the adoption (Fig. 2) and maintenance (Fig. 3) of the key personal factor themes. The bottom of each model outlines the interacting themes of each grouping and their influence on PA routine adoption and maintenance.

#### Step 3: behaviour change technique identification and intervention development

A proposal of the theoretically informed intervention is outlined in Table 3 and includes 26 BCTs with two additional BCTs (*body changes* and *credible source*) naturally occurring due to the sessions being delivered. The conceptual phases of adoption, routine formation, and maintenance were based on the inductive thematic analysis and behaviour change literature with the number of sessions informed by the interview transcripts. Of the included BCTs, 11 were recurrent, five were primarily coded to adoption, five to routine formation, and eight to the maintenance phases respectively. BCTs with a ‘behaviour’ and ‘outcome’ variant (e.g. goal setting) were listed as a single BCT and the final selection will be determined by patient’s preference when the intervention is delivered. For example, if the patient’s goal is to lose weight as a result of optimising PA, then the BCT would become goal-setting (outcome) as weight would be the outcome of consistently applying the behaviour.

**Table 3:** Theory-informed physiotherapy intervention outlining phases targeted by behavioural change techniques

Session Number	Targeted Phase	Specific BCTs	Reoccurring BCTs
1–2	Adoption	• Goal setting (behaviour and/or outcome) • Action planning (including implementation intentions) • Information on health consequences of performing the behaviour • Information on social and environmental consequences • Rewards (one of: Material, non-specific, social, self-reward, or outcome	• Review behaviour goals (and/or outcome) • Feedback on behaviour (and/or outcomes) • Self-monitoring of behaviour (and/or outcomes of behaviour) • Social support (unspecified) • Social support (practical) • Instruction in how to perform the behaviour • Modelling/demonstration of behaviour • Behavioural rehearsal/practice • Graded Tasks • Pharmacological Support • Body changes^a^ • Credible source^a^
3–4	Routine Formation	• Prompts/cues • Habit formation • Generalisation of a target behaviour • Adding objects to the environment (e.g. objects to facilitate behaviour)	
5–6	Maintenance	• Problem Solving • Behavioural Contract • Reduce negative emotions • Restructuring of social environment • Verbal persuasion about capability • Social comparison • Information about others’ approval	

*BCT* Behaviour Change Technique

^a^Denotes associated BCT occurrences naturally from delivering the intervention as planned

## Discussion

This is the first qualitative study to gain in-depth perspectives of the barriers and facilitators to physiotherapist prescribed PA during the treatment and post-discharge phases in patients with lower-limb OA. Inductive thematic analysis synthesised barriers and facilitators and themes and subthemes were collated into an a priori deductive framework which included three over-arching groupings: personal factors; treatment factors and; post-discharge factors. Subthemes were mapped to the TDF and results synthesised to outline the key domains to target for each theme. Thirteen TDF domains were identified and 26 BCTs were included in the proposed intervention, which represents the first theoretically informed physiotherapy intervention to target adherence to prescribed PA during treatment and post-discharge.

### Personal factors

Personal factors that influenced participants perceptions of PA adherence were consistent with previous literature, specifically: motivation [30, 33, 67], confidence [30, 34, 35, 39, 68], mindset [30, 34, 35, 39, 68] and experiences of arthritic symptoms [33, 34, 39] and/or PA [30, 34, 39]. Themes relating to personal factors were highly interactive and participants could be categorised into two broad profiles;

- Those with higher levels of arthritic symptoms and/or decreased physical capabilities, or who experienced symptoms as a result of PA reported less confidence, motivation and/or a negative mindset reported difficulty adopting and/or maintaining PA behaviours and required ongoing practical and social support [30].
- Participants who had internalised PA behaviours [16] or had high levels of social support reported feeling greater confidence, motivation and/or a positive mindset to adopt and maintain their PA post-discharge without additional need of support [30].

The TDF domain ‘*Beliefs about Capabilities’* was influential for all personal factor themes. This domain includes constructs such as of ‘self-confidence’, ‘empowerment’, ‘perceived competence’, and ‘perceived behavioural control’. These constructs relate closely with participant confidence, mindset, and the theoretical cognition of self-efficacy [69] which is a salient correlate of overall PA behaviours in patients with lower-limb OA [39]. Participants discussed that positive PA experiences increased their confidence and generated a positive mindset, which facilitated motivation to engage with prescribed PA. Therefore, self-efficacy may act as an antecedent to motivation in the behaviour change process [15] and BCTs that targeted the ‘*beliefs about capabilities’* construct (e.g. *instruction in how to perform the behaviour* and *demonstration of the behaviour*) were introduced early in the proposed intervention with several reoccurring.

### Treatment factors

Forming a PA routine was the key mechanism to promote PA behaviours during treatment. Participants felt that physiotherapists could optimise their routine by instructing prescribed PA and enabling participants’ to practice/rehearse within sessions [31, 32, 35, 36, 39, 68], slowly increasing PA in a graded manner, and providing feedback about PA or its associated outcomes (e.g. weight loss). Participants suggested that providing a demonstration of the behaviour (e.g. exercise sheet or video) and/or integrating self-monitoring of PA (e.g. pedometers or exercise diaries) into programmes would help regulate PA outside of the clinic during the treatment phase [70]. Interestingly, participants linked these BCTs to promoting confidence and purposeful PA. However, they are underutilised in clinical practice [70, 71] suggesting that physiotherapists may not be effective at utilising strategies which encourage PA adherence [72]. Therefore, these BCTs were introduced in sessions to and reinforced in sessions three-four to facilitate behavioural adoption and routine formation respectively.

The beneficial effects of a positive patient-physiotherapist relationship are in line with previous studies, as participants commented that encouragement [73], reassurance [36], and developing a strong personal connection enhanced adherence to PA [30, 74]. The proposed intervention therefore, utilises the reoccurring BCTs *social support (unspecified)* and *(practical)* respectively. Participants discussed how patient-centred treatment that involved collaboratively generated PA goals, led to feelings of empowerment and enhanced motivation to engage with PA [31, 75]. The use of a collaborative approach is associated with advanced clinical reasoning and expert physiotherapist practice [76]. Moreover, goal setting techniques with recurring review, were identified as important BCTs to promote PA adherence by patients with lower-limb OA [19], and were included in the adoption phase of the proposed intervention.

### Post-discharge factors

Several participants believed their physiotherapist could provide additional support post-discharge, as they perceived negative reactions from family/friends or peer PA groups [30, 38] due to their reduced physical capacity. As intervention time is limited for physiotherapists, it would be beneficial if community resources for post-discharge PA provision were integrated with the physiotherapy provider [34]. In line with previous studies, participants discussed that any post-discharge PA group needed to be exclusive to individuals of similar physical abilities [32, 34, 36, 38, 39, 68] and diagnosis to enhance relatedness [32, 36] and reduce feelings of anxiety or embarrassment [32, 39, 68]. Therefore, the proposed intervention incorporated BCTs relating to *problem solving*, and an indepth discussion on *how to structure the social environment* during the maintenance phase [28, 64, 65]. Community facilities primarily target those with decreased physical ability and lower income and need to be highly accessible [30, 31] and at minimal cost [19, 21]. However, particpants discussed that they found access to services highly problematic. Therefore, the proposed intervention included BCTs that alter the home environment, in order (to prompt routine formation and maintenance post discharge (e.g. using prompts and cues, or leaving Theraband in a prominent place to act as a reminder to their PA).

### Reflections on TDF mapping and BCT coding

With the exception of the motivation theme, TDF mapping was intuitive and consistent with all discussion points clarified by the two researchers. Motivation has a multi-faceted influence on PA adherence [30, 33, 67] that may change at different parts of the behaviour change process [16]. This is perhaps reflected in the refined TDF [28] which separated the original ‘goals and motivation’ domain [27] into the distinct ‘goals’ (preferred outcome), and intentions (determination to act in a certain way). While it is acknowledged that motivation is a distinct mechanism or process of action in current research [64, 65] further clarification of its definition within the TDF domains would likely increase consistency during mapping. These difficulties may also be linked to the topic guide design which was not informed by the TDF domains.

The TDF domains ‘environmental context and resources’ and ‘social influences’ were highly influential across both treatment and post-discharge phases. While the separation of themes into the personal factors, and treatment and post-discharge phases enabled some clarity of sequencing of BCTs, there was considerable overlap of domains across groupings. This suggests that behavioural adoption and maintenance have both unique and similar determinants.

Although the constructed matrix provided a useful tool, several BCTs did not overlap between studies and further work to establish agreement of the coding of BCTS from behavioural domains and mechanisms of action is required.

### Strengths and limitations

Strengths include the transparent methodology [77] to identify barriers and facilitators of PA adherence, which incorporated a valid and reliable framework to map them to determinants of behaviour change [24], and an extensively utilised strategy of identifying BCTs [28]. These strengths may enhance the interventions effectiveness at optimising adherence to PA. The study design facilitated an in-depth understanding of participant views and incorporating patient and public involvement further aided participants perspectives to be integrated during the intervention’s initial development.

A key limitation is that all participants were familiar with the person who recruited them, which may have influenced the findings. Furthermore, all participants were fluent English speakers of White British descent. While this reflected the hospital demographics, findings may not be generalisable beyond this population. In addition, participants recall PA levels were higher than those typically reported for patients with lower-limb OA [7]. Although the sample may have included only active participants, inconsistent and over-reporting of PA levels with patient reported methods of data collection is widely outlined in the literature [78], and perhaps strengthens the need for validated measures of objective PA in patients with lower-limb OA.

The theoretically informed physiotherapy intervention represents the initial proposal only. Prior to implementation, further data is required on the intervention’s feasibility to other stakeholders to clarify points on BCT mode of delivery, session lengths and frequency, and training needs of physiotherapists. Therefore, the feasibility and acceptability of delivering the intervention will now be tested in focus groups of physiotherapists working clinically prior to a phase two feasibility study.

## Conclusions

A proposed theoretically informed physiotherapy intervention to optimise PA adherence in patients with lower-limb OA during treatment and post-discharge was developed through a transparent and rigorous methodology. The intervention BCTs primarily target patients’ perceived beliefs about their capabilities, by developing a PA routine during treatment and facilitating appropriate psychosocial support and access to resources for PA maintenance post-discharge. The feasibility of delivery of the proposed intervention now requires evaluation.

## Supplementary Information

**Additional file 1.** Semi-structured interview topic guide.

**Additional file 2.** BCT assigned to matrix and groupings.

**Additional file 3.** Themes mapped to TDF across groupings.

## Data Availability

All data generated or analysed during this study are included in this published article [and its supplementary information files].

## Abbreviations

OA: Osteoarthritis
PA: Physical activity
TDF: Theoretical Domains Framework
BCT: Behaviour Change Technique
UK: United Kingdom
GP: General practitioner
COREQ: Consolidated criteria for Reporting Qualitative research
ROH: Royal Orthopaedic Hospital
SF HOOS: Short-form Hip Injury and Osteoarthritis Outcome Scores
SF KOOS: Short-form Knee Injury and Osteoarthritis Outcome Scores
SSC: Study Steering Committee
