# Supplementary material for: Utilising the perspectives of patients with lower-limb osteoarthritis on prescribed physical activity to develop a theoretically informed physiotherapy intervention
Source: BMC Musculoskelet Disord. 2021 Feb 8;22:155. doi: 10.1186/s12891-021-04036-8 (PMC7871381; doi:10.1186/s12891-021-04036-8)
Supplement: Supplementary file 1 — Additional file 1. Semi-structured interview topic guide. [file 12891_2021_4036_MOESM1_ESM.pdf]

Study title: STaying **A**ctive after **P**hysiotherapy in patients with **L**ower-limb **O**steoarthritis (STAPLO)

Semi-structured interview topic guide

Note: initial bullet: Main question; indented bullets: potential prompts

Introduction

- Thank you for agreeing to speak with me today about your experiences of the physiotherapy course that you have just finished
- I am going to begin by asking you a few questions about your background and levels of physical activity.
- I will then ask you a series of questions about your experiences of physiotherapy and whether it helped you be more physically active at the time and now that you have finished your treatment.
- I will finish by asking for your opinion on specific things that you think should be added to physiotherapy sessions that would help you to be as physically active as possible.
- It is important to note that there are no right or wrong answers to the questions and this interview should take about **30-60 minutes**.
- You do not have to answer or disclose anything that you do not want to, and you are free to end the interview or withdraw from the study at any point.
- If it is ok with you, **the interview will be audio recorded** to ensure that your key points are accurately documented. Everything you say will be **kept confidential, and the audio recording and transcription of what you say will be** stored in a safe and secure place
- You are also welcome to request a copy of your interview transcript if you would like to review it for clarification, to add to it, or to indicate that all or any part of it should not be used in this research project.
- Have you read the Participant information sheet and informed consent form?
- Do you have you any questions?

**At this point, the participant will be asked to sign consent form.*

Background data:

- How many times have you been referred to physiotherapy before? How many times was this for your osteoarthritis?
  ____
- Male/Female: ______________________________
- How old are you? ______________________________
- Do you remember how tall and heavy you were the last time you checked? ______________________________
- Where does your family originate from? ______________________________
  - Do you identify as being English?
- When did you exit education? ____________________________
  - Did you finish your GCSEs/O levels/A levels?
  - How old were you when you left school?
  - Did you go to University?
  - Did you finish your undergraduate degree/MSc/PhD?

*Note: Person with lower-limb Osteoarthritis will now be asked to complete the Pain Numerical Pain Rating Scale (below), the bothersome index (below), and either SFKOOS/SFHOOS questionnaire as appropriate.

- Please mark on the following scale the average amount of pain felt over the PAST WEEK in your most painful joint:

No pain possible — Worst pain

( ) 0 ( ) 1 ( ) 2 ( ) 3 ( ) 4 ( ) 5 ( ) 6 ( ) 7 ( ) 8 ( ) 9 ( ) 10

- In the last week, how bothersome has your osteoarthritis been in this joint?

  not at all ( ) slightly ( ) moderately ( ) very much ( ) extremely ( )

After Pain numerical rating scale, bothersome index, and SFKOOS/SFHOOS completed, interviewer will fill out 3-day Physical Activity Recall form (appendix 1) while asking questions on physical activity level.

I would like to know a bit more about the amount and type of physical activity that you engage in your everyday life.

- Were you employed in the last 3 day? _____
- How many days of the last three did you work? _____ days
- How many total hours did you work in the last three days? _____
- What two days do you consider your weekend days? __________

Sleep:

- Now I would like to look at the time you spent sleeping in the 3 days. By sleeping, I mean the time you went to bed one night and the time that you got out of bed the next morning. You may not necessarily have been asleep the entire time you were in bed. You may have been reading or watching television.
- Today is __________ so yesterday was __________. What time did you go to bed last night/the night before and get up this morning/morning before*?* (Note: This question will be repeated for each day, beginning with preceding day and going back for a total of three days)**.**

Physical Activity:

- Physical activity refers to any bodily movement that is performed by your skeletal muscles and requires energy, such as walking.
- Please think of the physical activities you have done in the past 3 days, starting with yesterday and going back. Please remember, this is a recall of actual activities for the past 3 days, not a history of what you usually do.
- We are not considering light activities, such as desk work, standing, light housework, strolling, and stop-and-go walking such as grocery or window shopping.

- We are interested in occupational, household, and sports activities that make you feel similar to how you feel when you are walking at a normal pace.

Intensity:

- I will ask you to categorize the intensity of each physical activity you do into one of three groups; moderate, hard, or very hard. The moderate category is similar to how you feel when you are walking at a normal pace. The very hard category is similar to how you feel when you are running. The hard category just falls in between. In other words, if the activity seems harder than walking but not as strenuous as running, it should go in the hard category

Segments of the day:

- I am going to ask you about the physical activities you engaged in during three segments of the day, which includes morning, afternoon, and evening. Morning is considered from the time you get up in the morning to the time you have lunch; afternoon is from lunch to dinner; and evening is from dinner until the time you go to bed

Today is __________ so yesterday was __________. Think about what you did yesterday morning/afternoon/evening. (Note: This question will be repeated for each day, beginning with preceding day and going back for a total of three days).

Potential Prompts:

Where were you? Think about what you usually do. Did you do anything unusual? Did you do any physical activity?

Duration:

How long did you do that activity? How much of that time was spent standing still or taking breaks?

Intensity:

Did that activity feel similar to how you feel when you are walking or running or is it somewhere in between?

How would you rate the intensity of that activity?

At end of first day recall:

Now I am going to ask you about activities you might do for building strength or improving flexibility. Strength activities include push-ups, pull-ups, sit-ups, lifting free weights, and using weight machines. Flexibility activities include holding stretches for several seconds or an activity like yoga.

For each subsequent day:

Did you do any strength or flexibility activities? How many minutes did you spend on each?

After all 3 days have been reviewed:

Are there any physical activities that you might have forgotten? Did you do any physical activity at work? any other recreational or sport activities? housework or gardening? Were there any other walks that you might have taken?

Compared to your physical activity over the past three months, was the last three days of physical activity more, less, or about the same? __________

Facilitators and barriers to physical activity and perceptions of physiotherapy

We are also interested in knowing about your thoughts on things that make it easier and harder for you to be generally physically active

- What makes it easier for you to be more physically active in your daily life?
    - How does it help?
    - What are the facilitators?
- What makes it harder for you to be physical active?
    - How do these make it harder to be physically active?
    - Any other barriers that you can think of?
- Has osteoarthritis effected your ability to be physically active?
    - If so, how?
    - How does this make it harder to be physically active?
    - So how physically active were you before you had osteoarthritis?
- With what you are doing now how, has physiotherapy made a difference to how physically active you are today?
    - Why? Why not?
    - Did you enjoy it? Why? Why not?
    - What might help improve it further?
    - Did you attend all the sessions? Why not?

During Treatment period

The next series of questions is dealing with how physiotherapy effected your physical activity levels while you were being treated. By that I mean your physical activity levels from your first appointment to when you were discharged including the time spent in between sessions.

- In your sessions, did the physiotherapist prescribe any exercises for you to do?
    - If so, what?
    - Did they make sure you were doing them correctly?
    - Did you find this effected your activity levels? Why/why not?
- Did the physiotherapist give you any further advice on how to be as physical activity as possible in between sessions?
    - What advice did they give you? E.g. setting goals, not doing too much when you had reduced pain, health benefits of physical activity, weight management, smoking etc

    - Did they give you any additional information? If so, what? e.g. sheet with exercises on it, TheraBand, activity diary, pedometer etc
    - Did it work? Why/ Why not?
- What made it difficult for you to make the changes to your physical activity in between your sessions that the physiotherapist suggested?
    - Why was this difficult?
    - What made it difficult?
- What helped make it easier for you to make the changes to your physical activity in between sessions that the physiotherapist suggested?
    - How did it make it easier?
    - What else could your physiotherapist have done to help you be more physically active?
    - Anything else you could think of?
- Can you think of any additional support, suggestions, or advice the physiotherapist could provide that would help you to be as physically active as possible in-between your sessions?
    - e.g. provision of a physical activity diary, education on appropriate levels of physical activity, knowledge about your condition, advice on pain management, setting goals, how to solve problems, further advice etc.
    - How would that help you be more physically active?
    - Anything else you can think of?

Post-treatment period

Now we are going to focus on your physical activity since you have been discharged from physiotherapy.

- Did the physiotherapist do anything or offer any advice to help you maintain your physical activity after you had finished your course of treatment?
    -
    - What did they do/suggest? E.g. reviewing your goals, education on health benefits of maintaining physical activity, use of 'buddy' to motivate, contact details of community support.
    - Did it work?
    - What was the most effective thing they did/suggested?
    - Why was this effective/ineffective?
- Is there anything that the physiotherapist suggested or did that makes it easier for you to be physically active now?
    - What did they say/do?
    - How does it make it easier for you to be more physically active?
- Is there anything that the physiotherapy suggested or did that makes it more difficult for you to be physically active now?
    - What were they?
    - How do they make it more difficult to do your physical activity?

- Can you think of any additional support, suggestions, or advice the physiotherapist could provide that would help you to be as physically active as possible now your sessions have ended?
    - e.g. reviewing your goals, education on health benefits of maintaining physical activity, use of ‘buddy’ to motivate, contact details of community support .etc.
    - How would that help you be more physically active?
    - Anything else you can think of?

Focused questions on session delivery preferences

Ok now look at your programme overall, now you have been through it from start to finish

- Would there have been anything else you would have wanted included in the physiotherapy programme which would help you be as physically active as possible?
    - What?
    - How would this help with your activity levels?
- What are the most important things to include in the physiotherapy sessions to help you be as physically active when you first start physiotherapy treatment in between your sessions?
    - E.g. More education around your condition, use of physical handouts, diary, pedometer, advice on rearranging your physical space at home, incorporation of peer support etc
    - How would they help?
    - Anything else you can think of?
- What are the most important things to include in the physiotherapy sessions to help you be as physically active as possible now you have finished your sessions?
    - E.g. Follow up texts or telephone calls, referral to a community group? Further follow up with the physiotherapist? Dedicated phone line that you can call?
    - How would they help?
    - Anything else you can think of?

- How many physiotherapy sessions do you think you would require before you feel confident that you could maintain your physical activity levels (i.e. self-manage)?
- How would you like these sessions delivered?
    - *How long/often/ specific time of the day*
    - In person?
    - Could any be done over phone/skype?
    - Are there any other things you can think of which you would make these sessions better?

Conclusion:
Thank you for speaking with me. I enjoyed our discussion. Any other points you would like to make on any of the topics we discussed?

Appendix 1: 3-day Physical Activity Recall Form (filled out by interviewer during semi-structured interview)

	Days			
Sleep	Time in bed			
	Time out of bed			
Morning	Moderate			
	Hard			
	Very Hard			
Afternoon	Moderate			
	Hard			
	Very Hard			
Evening	Moderate			
	Hard			
	Very Hard			
	Strength			
	Flexibility			
Total mins per day				
