# Supplementary material for: Utilising the perspectives of patients with lower-limb osteoarthritis on prescribed physical activity to develop a theoretically informed physiotherapy intervention
Source: BMC Musculoskelet Disord. 2021 Feb 8;22:155. doi: 10.1186/s12891-021-04036-8 (PMC7871381; doi:10.1186/s12891-021-04036-8)
Supplement: Supplementary file 2 — Additional file 2. BCT assigned to matrix and groupings. [file 12891_2021_4036_MOESM2_ESM.pdf]

Additional File 2:

Behaviour Change Technique and Domains Matrix

	TDF DOMAIN	KN	SK	SPRI	BOCAP	OP	BOCON	REIN	INTENT	GOAL	MAD	ECR	SOCI	EM	BEHR
**BCT**															
**1.1 A**			X						XX	XXX					X
**1.2**			X		XX				X	X	X			X	
**1.3**			X						X	XXX					
**1.4**										X					
**1.5**										XX					
**1.6**										X					
**1.7**										XX					
**1.8**									X Y	X					
**1.9**									Y						
**2.1**			X												
**2.2**		X			X		X								
**2.3**			X		X		X				X				X
**2.4**															X
**2.5**			X												
**2.6**		XY													
**2.7**					X		X								
**3.1**				X	X				X	X			XXX		
**3.2**				X	X				X	X		X	XXX		
**3.3**				X	X				X	X			XY	Y	
**4.1**		X	X		X										
**4.2**		XY													X
**4.4**															
**5.1**		X XX					X		X						
**5.2**							X Y								
**5.3**		X X					XX Y		X	X					
														Y	
**5.5**							X Y								
**5.6**							X Y							Y	

**6.1**	X	X							XX		
**6.2**									XX		
**6.3**									XY		
**7.1**							XX	XX			
**7.2**								Y			
**7.5**								X			
**7.8**				Y							
**8.1**	XXX	XX									
**8.2**											X
**8.3**	Y										
**8.4**	Y										
**8.6**	X										
**8.7**	XXY	XX			X	X					
**9.2**	X	X	X Y		X	X					
**9.3**			X Y								
**10.1**			X	XX							
**10.2**				XX							
**10.3**	X			XY	X	X					
**10.4**				XY					XY		
**10.5**				X							
**10.6**	X			XX	XX	X					
**10.7**				X							
**10.8**			X	XX							
**10.9**				Y							
**10.10**			X	X							
**10.11**			Y	Y							
**11.2**										XXY	X
**11.3**							X				X
**12.1**								XXX			
**12.2**								XXY	Y		
**12.3**								XY			
**12.5**								XX			
**12.6**	Y										
**13.1**									Y		

**13.2**														
**14.1**							Y							
**14.2**							Y							
**14.3**							Y							
**14.4**							Y							
**14.6**							Y							
**14.7**							Y							
**14.8**							Y							
**14.9**							Y							
**14.10**							Y							
**15.1**				X Y	Y	X		X	X					
**15.3**				X Y										
**15.4**				X X										
**16.1**						Y								
**16.2**						Y								
**16.3**						Y						Y		

Legend

BCT: Behaviour Change Technique; KN: Knowledge; SK: Skills; SPRI: Social/Professional Role and Identity; BOCap: Beliefs about Capabilities; OP: Optimism: BOCON: Beleifs about Consequences. REIN: Reinforcement; INTENT: Intentions; GOAL: Goals; MAD: Memory, Attention and Decision Processes; ECR: Environmental Context and Resources; SOCI: Social Influences; EM: Emotion; BEHR: Behavioural Regulation; BCT number as per Michie et al., 2013.

Red: Michie et al., (2008)

Green: Cane et al., (2015): all significant or borderline included form top down task: X = commonly used; Y = less common

Blue: The theory and techniques tool synthesising work by Carey et al., (2019), Connell et al., (2019) and Johnson et al., (2020). From https://theoryandtechniquetool.humanbehaviourchange.org/tool

Coding Decisions

Michie 2008:

- Goal setting and Monitoring separated into behaviour and outcome
- Rewards; incentives: coded as 10.3/10.6 Non specific
- Increasing skills: coded as problem solving and pros and cons (decisional balance)
- Stress management: coded as Reduce Negative Emotions
- Coping skills: coded as problem solving
- Rehearsal of relevant skills: coded as behavioural practice/rehearsal
- Role play: coded as demonstration of target behaviour
- Implementation: coded as action planning
- Prompts, triggers, cues: coded as Prompts and cues
- Environmental changes: coded as 12.2 Restructuring the social and physical environment; 12.5. Adding objects to the environment
- Social Processes of encouragement, pressure, support: coded as Social support (all)
- Persuasive communication: coded as Verbal persuasion about capability
- Information regarding behaviour, outcome: Information about health consequences, Information about social and environmental consequences
- Personalised message: not coded
- Modelling/demonstration of the behaviour: coded as demonstration of the behaviour
- Homework: not coded
- Personal experiments: coded as behavioural experiments
- Experiential: not coded
- Feedback: coded as feedback on behaviour; feedback on outcomes of behaviour
- Use of imagery: not coded
- Perform behaviour in different setting: coded as generalisation of the target behaviour
- Shaping of the behaviour: coded as Instruction in how to perform the behaviour
- Motivational interviewing: coded as Social support unspecified
- Relapse prevention: coded as problem solving
- Cognitive restructuring: coded as Framing/reframing,

- Relaxation: coded as body changes
- Desensitisation: not coded
- Time management: not coded
- Identify/prepare for difficult situation/problems: not coded

Cane et al., 2015:

- Vicarious reinforcement: coded as vicarious consequences
- Incentive: coded as all of 10.1. Material incentive (behaviour) 10.5. Social incentive 10.6. Non-specific incentive 10.7. Self-incentive 10.8. Incentive (outcome)
- Self-assessment of affective consequences: coded as Monitoring of emotional consequences

Initial Behaviour Change Techniques coded to Groupings

Grouping	Coded from	Potential BCTs
Personal factors	Matrix	1.2, 2.2, 2.3,2.6, 2.7,.3.1,3.2,3.3, 4.1,4.2,5.1,5.2,5.3,5.4, 5.6, 6.1, 8.1, 8.3, 8.4, 8.7, 9.2, 9.3,10.11, 11.2, 15.1, 15.3, 15.4, 16.1,16.2,16.3
	Transcripts	1.4, 2.2, 2.3, 2.4, 3.1,3.2, 4.1,6.1, 8.1, 8.7, 11.1
Treatment Phase	Matrix	1.1, 1.3, 1.4 [a], 1.5, 1.7, 2.2 [a], 2.4, 2.6, 3.1 [a], 3.2 [a],3.3 [a], 4.2, 5.1, 6.1 [a], 6.2, 6.3, 7.1,7.2, 7.5,7.8, 8.1 [a], 8.3, 8.4, 8.7 [a], 10.1, 10.2, 10.3, 10.4 [a], 10.5,10.6,10.7, 10.8, 10.9, 10.11, 12.1, 12.2, 12.3, 12.5, 12.6, 14.1, 14.2, 14.3, 14.4, 14.6, 14.7, 14.8, 14.9, 14.10, 13.1, 16.3
	Transcripts	1.1, 1.3, 1.4 [a], 5.1, 5.3, 4.1 [a], 6.1 [a], 8.3, 8.6, 8.7 [a], 10.4, 10.6, 12.5
Post-Discharge Phase	Matrix	1.2, 2.2 [a], 2.3 [a], 2.7, 3.1 [a], 3.2 [a], 3.3 [a], 4.1 [a], 6.1 [a], 6.2, 6.3, 7.1,7.2, 7.5, 8.1 [a], 8.7 [a], 9.2, 10.4 [a], 12.1, 12.2, 12.3, 12.5, 13.1, 15.1, 15.3, 15.4, 16.3
	Transcripts	1.2, 1.8, 11.2 [a], 12.2, 15.1

Legend: BCT: Behaviour Change Technique; [a]: denotes commonly identified overlapping Behaviour Change Techniques
