# Supplementary material for: Utilising the perspectives of patients with lower-limb osteoarthritis on prescribed physical activity to develop a theoretically informed physiotherapy intervention
Source: BMC Musculoskelet Disord. 2021 Feb 8;22:155. doi: 10.1186/s12891-021-04036-8 (PMC7871381; doi:10.1186/s12891-021-04036-8)
Supplement: Supplementary file 3 — Additional file 3. Themes mapped to TDF across groupings. [file 12891_2021_4036_MOESM3_ESM.pdf]

Additional File 3: Themes and subthemes with examples quote and corresponding Theoretical Domain Framework (TDF) Domain

Table 1: Personal Factors

**Theme/**sub-theme	**Example of quotation (participant no; 'quotation')**	**TDF Domains**	**Participant Numbers**	**Conflicting Belief (y)**	**Strong Belief (y)**
**Motivation**					
Seeing progress	(4) 'You need to feel that you are making progress, and as I have, this is definitely affected my motivation and made me do my exercises.'	Skills<br>Beliefs about capabilities<br>Intentions<br>Reinforcements	1,2,3,4,7,8,<br>9,11,13		Y
Having a purpose	(2) 'I suppose it's motivation, it's having something to do and the need to get out because I make that, when I don't have to go out, I make a point of going out for a walk and having that 11:00 cup of coffee. I think it's that motivation'	Goals<br>Intentions	2,4,8,10		
Wanting previous level of function	(1) 'Because I used to be ever so quick, you know what I mean... For me it was more of like, to get physically back to where I wanted to be.'	Goals	1,2,7, 8,9,<br>10,11,12,13		Y
Fear of functional decline/ need for invasive treatments	(9) 'I know that I'm going to carry on doing it because I've got a very simple goal: over my dead body am I going to need a hip replacement…it's an absolute motivator for me. I do not want to go down the read of needing opioids, steroid injections or a referral for surgery. I absolutely loathe hospitals.'	Goals<br>Intentions<br>Emotions	1,2,7,9,10,1<br>1		Y
Wanting to reduce or control symptoms	(7) 'What made me do them was because I thought, "I've got to get rid of this pain and that, I'll have to do them."<br><br>(11) 'regards the arthritis, I was going to rule it, it wasn't going to rule me…there's two ways of dealing with it. It could rule me and say right I can't do that I've got this, or I	Goals<br>Intentions	1,2,3,7,8,9,<br>10,11		

	could say right, I’m not letting this stop me from doing what I want to do and do my exercises.’				
Enjoyment of physical activity/Exercise<br>External sources of motivation	(8) ‘For me, the motivation would just have been to carry on doing the things I wanted to do which was ride, walk ideally, and that’s changes more to cycling now.’	Goals<br>Intention<br>Emotion	2,3,4,8,9,<br>12,13	Y	Y
Using the physiotherapist as a motivator	(9) ‘I wanted to show the physiotherapist I could do the exercises properly’	Knowledge<br>Skills<br>Intentions<br>Goals (goals)<br>Social Influences	6, 8, 9,10		
Using items as a motivator (TheraBand, pedometer)	(6) because of the steps, the amount of steps that you do a day, you sort of keep up and you’d look at it and you’d think, right okay, I’ve done 2,000 steps today, tomorrow I want to do 2,050, I think it would push yourself to do more’	Intentions<br>Environmental context and resources<br>Self-regulation<br>Reinforcement	3,6,13	Y	
Guilt	(2) ‘I feel guilty if I do not do my exercises.’	Beliefs about consequences<br>Emotion	2,8		
Personal responsibility for own PA/Exercise	(12) ‘You’ve got to give it a go, at the end of the day if you want it to work; again, that’s just personal preference. If you want it to work, you’ve got to put a bit of effort in yourself.’	Intentions	2,4,7,8,9,10<br>,<br>11,12		Y
Excuses to not do PA/Exercises	(6) ‘In a way I sort of use it as a bit of an excuse, I know he can’t walk as well as he used to’	Intentions	1,6		
**Confidence**					
Realisation that PA reduces symptoms and improves function	(13) ‘By doing the exercises…it made me realise, I suppose, that I could do something about it and it gave more confidence’	Knowledge<br>Skills<br>Beliefs about capabilities<br>Beliefs about consequences<br>Reinforcement	1,2,3,4,7,8<br>11,12,13		Y

Increased feelings of capability from doing PA	(1) 'You've gone through the exercises and that and I don't know, you just feel so much more capable of doing everyday things'	Knowledge<br>Skills<br>Beliefs about capabilities<br>Optimism<br>Reinforcement	1,3,4,8,9,10<br>11,13		
Confidence in skills to maintain activity	(2) It's all about maintaining, I think. What do I need to maintain? It's being confident that I've got the tools (exercises) necessary	Knowledge<br>Skills<br>Belief about capabilities<br>Beliefs about Consequences	2,3		
Fear-avoidance	(3) 'The noises in my joints are horrendous. It makes me nervous to be active.'<br><br>(9) 'Most of the time I don't need a stick, but the thing is, when…I can have times when this hip is so painful and my leg feels as if it's going to support me. The stick isn't to help me walk properly. The stick is to give me confidence that, if I get that feeling in my leg, that I am not going to fall over.'	Knowledge<br>Skills<br>Beliefs about capabilities<br>Beliefs about consequences<br>Optimism<br>Environmental context and resources	2,3,7,9,13	Y	Y
**Mindset**					
Psychological effects of improvement of symptoms'	(2) 'It was quite a psychological boost to feel that one part of me, physically, could improve so quickly and so helpfully and I wasn't on the downward spiral. Psychologically that is so important, it really is.	Knowledge<br>Skills<br>Beliefs about capabilities<br>Optimism<br>Beliefs about consequences<br>Reinforcement<br>Emotion	2,6,7,8		Y
Mental and emotional resources	(8) 'Maybe that was part of why it was hard to do myself, but part of my motivation for stopping treatment was because I just felt like I hadn't got any more, you know, space to do stuff really.'	Beliefs about Capabilities<br>Optimism<br>Memory attention and decision processes<br>Environmental Context and resources<br>Emotion	1,8		Y

Comfort zone	(1) ‘It was quite difficult at first. I think I was out of my comfort zone being in a room with people you didn’t know, but then after the first week, when I went back the second week, I found it much easier.	Beliefs about capabilities<br>Optimism<br>Environmental context and resources<br>Social influences<br>Emotion	1,6,8,9		
**Arthritic symptoms**					
My pain stops me from being active	(12) ‘Personally, I’m thinking, as I am at the moment, because I have been in quite a bit of pain I haven’t been as active now because I want to come home and rest.’	Knowledge<br>Beliefs about capabilities	1,2,3,4,7,8, 9<br>10,11,12,13	Y	Y
Pain stops me doing my exercises	(7) I was lying on the bed and I mean, that was painful because my hip was playing me up at the time and I couldn’t do them’ (exercises)	Knowledge<br>Beliefs about capabilities	4,5,7,8,9,10<br>11	Y	Y
Pain affecting mood and reducing PA	(2) I was getting very depressed with the pain I was in and starting to not go out and starting to not walk. I felt this is it.’	Beliefs about Capabilities<br>Optimism<br>Emotion	2,7,12		Y
Pain affecting enjoyment of PA	(4) ‘because obviously if it doesn’t hurt so much (laughter) you enjoy it a bit more’	Optimism<br>Emotions	4,8,10,12		Y
Other symptoms in joints (apart from pain)	(12) ‘I am stiff in the morning because of my arthritis.’<br>(3) ‘The stiffness controls me while I’m playing golf.’<br>(2) my flexibility has gone; I’m just generally walking with a limp and so on	Beliefs about capabilities<br>Beliefs about consequences	2,3,8,12		

Pharmacology reducing symptoms to enable PA	Injection therapy: (10) When my knee had the injection, it just lessens and takes the pain away. Without the injection, my knee aches constantly though the day, aches, pains, I'll get a sudden jolt like electric jolt of pain<br><br>Oral Medication: (3) When I play golf, I take a painkiller, I must admit. I don't because I'm not into painkillers. But I take one before I leave, co-codomol. It might be one, it might be two and then that kicks in by the time I'm up and running and that gets me round then I'm all right<br><br>Topical Gel: (8) 'If I do walk, I have to use a gel, an ibuprofen gel, and if I do that, I can walk without too much discomfort.'	Knowledge<br>Environmental context and resources	1,3,4,8,10,<br>12		Y
**Experience of PA**					
Benefits of activity (well-being/general health)	(2) 'I know that for my health I need to get out, fresh air, walk, so I make it like, it's almost as important as having lunch, do you know what I mean? That's my daily exercise and I make sure I get it in. If I don't, I miss it.'<br><br>(2) 'Once I'm active there's less problems really. The more active I am, the better I am, I know that'	Skills<br>Beliefs about Capabilities<br>Beliefs about consequences<br>Reinforcement<br>Emotion	2,4,10,12,1		Y
Effects of PA (mind-set)	(2) 'The exercises made me more positive. Instead of thinking, I can't do this, I started, it's psychological, I can do this. It's not going to be a burden; it actually could be a pleasure.'	Skills<br>Beliefs about Capabilities<br>Beliefs about consequences<br>Reinforcement<br>Emotion<br>Optimism	1,2,11		Y
Effects of PA (arthritic symptoms)	(4) 'I think it's to do with less pain from the exercises, because at one time it was like a knife going through my knee, as I was trying to put the weight on the right knee. So, it's less pain, and more strength.'	Skills<br>Beliefs about capabilities<br>Beliefs about consequences<br>Reinforcement	1,2,4,6,7,9,<br>10,11,12,13	Y	Y

	(10) 'It was just that one exercise when my knee actually didn't like and, well stiffened. It wouldn't bend. I couldn't walk. Something happened. So, I don't know what happened to my knee, but I just could use it, I couldn't drive, I couldn't walk.'			
Effects of PA (function e.g. stairs, walk, balance)	(11) 'I used to go upstairs one at a time, you know. One, one, one and now I can go one, two, one, two.'	Skills<br>Beliefs about capabilities<br>Beliefs about consequences<br>Reinforcement	2,4,7,11	Y
Increased symptoms from too much PA	(3) 'I can only do what I'm doing. I wouldn't try to do something above that because I've just got to pay the price for it, so…I just do what I know I'm capable of doing without making myself suffer really.'	Intentions<br>Beliefs about capabilities<br>Beliefs about consequences<br>Reinforcement	1,3,4,5,7,9, 10,12,13	Y
**Previous levels of Physical activity**				
Identifying as physically active	(13) Being very physical, has always been part of my USP, I suppose, as a person it's been quite difficult to come to terms with the fact that I can't do that anymore.'	Skills<br>Social or professional role or identity<br>Beliefs about capabilities	8,9,10,12,1 3	Y
Adjusting expectations	(12) 'I think sometimes you've got to have a hard conversation with yourself and say look, you've got this, with best will in the world you may never get to the stage that you were before'	Beliefs about capabilities<br>Intentions<br>Goals	7,12	
Frustration at loss of function	(12) 'things I used to be able to do, which I think was really easy, even walking now, long walking, I'm starting to hurt quite a bit in my leg. I used to walk for miles. It's frustrating because I know what I could do and what I want to do, but I can't.'	Beliefs about Capabilities<br>Optimism<br>Emotion<br>Reinforcement	7,12	Y

Table 2: Treatment Factors

**Theme/**sub-theme	**Example of quotation (participant no; 'quotation')**	**TDF Domains**	**Participant Numbers**	**Conflicting Belief (y)**	**Strong Belief (y)**
**Access**					
Other obligations (work)	(10) 'And when I was working and I'd got the hectic schedule and so much going on in my life, it did make it more difficult. And it made it difficult to get to the actual physiotherapy session, because as I say, they give you a time and the you think, 'oh, no I can't do that one, I'm in a lesion that day.' Or they'd change my timetable at work, and I was having to try and swap things, it was a nightmare.'	Environmental context and resources	6,10,11		
Appointment flexibility	(13) 'I thought they were really flexible actually. My first appointment was 7:40, so I did it before work and it was utterly fine. All my other ones have been early-ish, so I could get into work. I didn't realise that that was a thing, so that's quite useful.'	Environmental context and resources	1,12,13		
Number of sessions	(2) 'You go there and then you start doing the physio and you just get to that, she says to have five and I actually, and they gave me six, which I was really, you know, thankful for. I would like to say I could have done with a few more.'	Environmental context and resources	1,6,10		
Duration of sessions	(7) 'I was called in, my husband came with me and he was sitting where I met you, he was sitting there. And I come out and he says, "Where are you going?" I said, "I'm finished." I do think they should really spend a bit longer.'	Environmental context andresources	5,7,12		Y

Long referral process	(12) ‘Excuse me, I’m sitting here with pain every day, 24/7 for the last six months and I’m coming back and badgering you saying it’s still hurting. It took quite a while to get to that stage as well.’	Environmental context and resources	2,9,12,13		
**Value of sessions**					
Increased motivation	(2) ‘Every time I attended one of those sessions, I came out feeling more motivated and more hopeful because nobody said, “Go away, you’re old, live with it.’	Optimism<br>Reinforcement<br>Emotion<br>Social influences<br>Environmental context and resources	1,2,5,6		
Empowered/ control of symptoms	(13) The experience has had has been really, It’s been really kind of empowering, because I know how to take control, of what I’m working with<br><br>(9) When she first said those words “osteoarthritis”, my heart absolutely sank because I know an awful lot about arthritis, but she was able to make me feel that I was in control of it	Knowledge<br>Beliefs about capabilities<br>Social influences<br>Environmental contexts and resources	2,3,5,9,13		Y
Too much talking	(12) ‘There’s been a lot of turning up at appointments, talking about something and I’ll go away and just, okay, you’ve talked about it, but you’ve actually done nothing for me.’	Environmental context and resources<br>Social influences	3,5,7,9,12		Y
Physiotherapist didn’t do anything	(5) ‘I just don’t understand that, when I came out, I spoke to them, who I seen in the…she said “It’s a load of bloody rubbish, they didn’t do nothing.” And they just said, Well, that’s it now…” I thought bloody hell.	Environmental context and resources<br>Social influences	3,5,7,12		Y
Doing exercises in the sessions	(7) ‘But I don’t do the exercises there. Not now. That’s what makes me think, “Is it worth coming all the journey?” I might as well do them at home. And I do them better at home. Because	Skills<br>Environmental context and resources<br>Social influences<br>Reinforcement	6,7,9,12	Y	

	I'm only coming now to talk to her.'<br><br>(12) One of the things I was doing was stretching, lying down and stretching the leg out…coming away from that session is like, oh, that's great. I walked out of here and I thought, oh, I really felt a sense that it eased up.'	Intentions<br>Emotions		
**Relationship with physiotherapist**				
Getting to know patient as an individual	(13) 'She got the lay of the land, on a personal level pretty quickly. She identified through an examination and also through conversation, that I was quite active anyway.'	Social influences	1,2,4,6,7,8,9,13	
Collaborative relationship	(8) 'there's something about the engagement in that process, engaging in your own recovery and feeling that some else is also engaged in that and it's not just their job, you know…'	Optimism<br>Social influences	1,2,4,8,9,10,13	Y
Familiarity	(7) 'Yeah at least I know she's there. But I think for my hip, I think the more I do the exercises, but I feel safer if it's with (name).'	Social influences<br>Beliefs about capabilities<br>Reinforcement	7,10,11,12	
Physiotherapist was motivating	(4) 'He is absolutely fantastic in all ways, because I have seen other physios before and found them useful, be he is so motivating and so kind….And I think that his input has been particularly important, because there are other physios I've seen that I thought oh.'	Optimism<br>Social influences	1,2,4,6,7,10,<br>12,13	
the physiotherapist was friendly/ encouraging	(2) 'They were absolutely brilliant. I cannot think of anything negative about them. They were encouraging, honest, personable and I couldn't	Optimism<br>Social influences	1,2,4,6,8,10,11	Y

	wish for better people.'			
Encouragement from the physiotherapist helps with mental well-being	(1) 'I think having encouragement from them (is the most important thing)…I think sometimes when you've had things like that, it's not just physically, its mental as well.'	Optimism<br>Social influences	1,4,8,6,10,11	
Communication	(2) 'I suppose it's down to communication. He would ask what the problem was and then he would offer some solution… they can remember some of the conversation we had last time and they speak to me as a human being and there's humour and they're just brilliant.'	Optimism<br>Social influences	2,4,8,9,13	
Attitude of healthcare professional	(10) 'He has a bit of an attitude…he made me feel like I was wasting his time…I'm quite stoic, I will put up with pain and I was thinking, maybe I'm not putting up with pain as much as I should. So, I wouldn't go back. And I was in absolute agony and my husband was pleading with me, "Go back, go back." And I'm like, "No, no." Because they just made me feel that big'	Optimism<br>Social influences	3,10,13	
**Person centred Treatment/ Personalised Treatment plan**				
Patient centred treatment	(8) 'I suppose to explore what you enjoy and to try and make that into thing that, you know, your regime is built around or your exercise is built around. I don't know, to not have a formula of easy exercises you do if you got an arthritic knee, to personalise it really.'	Knowledge<br>Skills<br>Goals<br>Social influence	2,4,8,9,12,13	Y

(Re) Assessment of joint	(2) 'I had (name) who figured out what was wrong with me and then he realised the back of my leg was all very, very stiff because of what has happened.'<br><br>(7) 'She didn't even check my knee. She did once, check my knee, the bending on it and all that. But you can't see anything.'	Knowledge<br>Skills<br>Beliefs about capabilities<br>Social influence<br>Reinforcement	1,2,4,7,8,9,10,12	Y	Y
(Re) Assessment of function and overall ability	(12) 'It would have been good to say, right, let's do a session where we can see, actually can you do this now? Can you bend over and pick up that item up, can you stretch, cam you walk up and down, what are you looking like?....you wouldn't fix a car and then not take it out for a test drive to see it's fixed. You wouldn't say, right, I've fixed this, just leave it sitting in the garage and expect that it's worked. You've got to take it for a test drive and see it's working.'	Knowledge<br>Skills<br>Beliefs about capabilities<br>Social influence<br>Reinforcement	3,4,5,7,8,12		Y
Assessment of exercises/PA	(2) 'he checked and asked me to do the exercises as he prescribed, to make sure I was doing it properly…this made me feel much more positive.'	Knowledge<br>Skills<br>Beliefs about capabilities<br>Optimism<br>Social influence<br>Reinforcement	1,2,4,7,8,9,10,12		Y
Feedback on exercises	(9) 'She showed them to me and got me to do them in each session, and critiqued what I as doing and made suggestions for how I could do them better.'	Knowledge<br>Skills<br>Beliefs about capabilities<br>Social influence<br>Reinforcement	1,6,7,8,9,10,12,13		

Instruction and demonstration	(11) 'Well, really, I'd say the exercises and showing me how to do them (most important thing that the physio did) they need to ensure I know how do them.'	Knowledge<br>Skills<br>Beliefs about capabilities<br>Social influences	2,3,6,9,11,12,13		Y
Explanation of what PA/exercises are supposed to do	(3) 'I don't know what else, other than an operation, they could do that would make it better. I don't know. I don't know whether the exercises, or what different sort of exercises would do it. Like I say, I think, what I was doing was to strengthen the muscles in the knees and thighs, and when you do that it pulls and it's all strength muscles and I don't know whether, in my particular case, the muscles needed strengthening'<br><br>(9) 'She was also very good in terms of explaining why the exercises were important and what they were designed to do.'	Knowledge<br>Skills<br>Beliefs about capabilities<br>Social influences	3,6,9,11	Y	
Having a detailed plan	During treatment (8) 'also making a plan. I do quite like having a plan or a to-do list for something which might incorporate activity and exercise.'<br><br>Maintenance: (2) 'I think it's just, the word 'schedule' comes to mind…if they just said this amount of walking every day, this is a place you can go to a couple of times a week to get the stretches in, then this would go to maintain where you are.'	Knowledge<br>Skills<br>Goals<br>Behavioural Regulation	1,2,6,7,8,9,12,13		Y
Written or visual instructions	(1) 'If they had to put it down in black and white, this is what you need to really do and then you	Knowledge<br>Reinforcement<br>Skills	1,3,5,6,12,13		

	can progress after a week two or three. I think to have something in black and white really.'<br><br>(13) 'They were quite simple exercises initially and honestly, the second set of exercises, she didn't write down and I couldn't remember one of them. A visual aid is quite useful just to…yeah particularly if you're unfamiliar with the routine of it and how far you can go.'	Goals<br>Behavioural Regulation		
Detail on Dose/frequency etc	(9) 'One of the things that really confused me when I first started doing the exercise was, I said, "(Name), how many repetitions should I do?" She said, "Do whatever you're happy with."….I think they should be saying…"Look, to keep your hip in a reasonable state, you need to be doing 10 repetitions a day," something like that.'	Knowledge<br>Skills<br>Goals<br>Behavioural Regulation	2,8,9,12,13	
Level of PA/exercise	(4) 'I think to assess your ability and set it (exercises) at the right level to start with because if they set it too high, you're not going to do it.'	Knowledge<br>Skills<br>Beliefs about Capabilities<br>Goals<br>Behavioural Regulation	1,4,6,8,9,10,11,<br><br>12,13	Y
Gradual Progression	(13) 'I suppose it's how that relationship kind of grows in terms of, there's some exercise, some simple exercise to start, then it gets a bit more complicated. Then is gets more complicated and then she's saying, "keep doing that and now and then go for a swim." It's kind of that build up, I suppose of instruction.'	Knowledge<br>Skills<br>Beliefs about Capabilities<br>Goals<br>Behavioural Regulation	1,2,4,8,9,12,13	Y
New exercises	(4) 'Every time I go back, and he gives me a new exercise, I'm like, "I'm going to do new exercises." And I have done them, I can honestly say I have done them.'	Knowledge<br>Skills<br>Beliefs about Capabilities<br>Intentions	4,12,13	

		Behavioural Regulation		
Knowledge about OA	(13) 'I like to know, it is about education, I want to know what the problem is. In terms of what I can and can't do, what I might expect, what the prognosis is, what is this pain that I'm experiencing, what is it? I'm very familiar with how it feels, but what actually is it? What's causing it? What's the problem?'	Knowledge	2,9,12,13	Y
Education about OA	(13) 'I like to know, it is about education, I want to know what the problem is. In terms of what I can and can't do, what I might expect, what the prognosis is, what is this pain that I'm experiencing, what is it? I'm very familiar with how it feels, but what actually is it? What's causing it? What's the problem?'	Knowledge	1,2,5,10,12,13	Y
Advice about OA (treatment options, none-physiotherapy management)	(9) 'It sounds daft to say that I need to be told what sort of exercise to avoid, because you would think that, if it was an exercise you should avoid, it would be blooming painful, but I know that it's quite possible to do something that's very bad for your joint. It doesn't actually cause pain at the time. It causes pain afterwards, so some general advice about good joint self-care, things to be thinking about.'	Knowledge<br>Beliefs about Capabilities	1,2,5,9,12,13	
**Routine**				
**routine effect on PA adherence**	(2) 'the word 'schedule' comes to mind. If they just said, this amount of walking every day, this would help me do my exercises.'	Knowledge<br>Skills<br>Beliefs about capabilities<br>Reinforcement<br>Intentions<br>Goals	1,2,4,5,6,<br>8,10,13	Y

		Behavioural regulation	
**Integrating PA/exercise into daily life**	(9) ‘You see, the thing is I combine lots of things, so I’ll be standing in the kitchen, I’ll be… I’ll do one of the exercises, which probably takes two minutes, two/three minutes-ish, but then I’ll stand and finish the washing up. Then I might do another of the exercises.’	Knowledge<br>Skills<br>Reinforcement<br>Intentions<br>Goals<br>Environmental contexts and resources<br>Behavioural regulation	2,3,5,6,7,9,<br><br>10,11,12,13
**Ways to promote routine**	(8) ‘something like a thing where you had to score every day even what your level of pain was and what exercise you’d done, something like a diary or something that you were in a habit… because if you establish a habit, don’t you, the likelihood is that you’ll keep doing it.’	Knowledge<br>Skills<br>Goals<br>Reinforcement<br>Behavioural regulation	2,7,8,10,11,<br><br>13
**Effects of routine (well-being/ general health)**	(2) ‘I think it’s routine/discipline because I know that for my health I need to get out, fresh air, walk, so I make it like, it’s almost as important as having lunch, do you know what I mean? That’s my daily exercise and I make sure I get it in. If I don’t, I know I miss it.’	Knowledge<br>Skills<br>Beliefs about consequences<br>Reinforcement<br>Intentions<br>Goals<br>Behavioural Regulation	1,2
**Effects of routine (motivation)**	(2) ‘I suppose it’s motivation, it’s having a routine and something to do and the need to get out because I make that, when I don’t have to go out, I make a point of going out for a walk and having that 11:00 cup of coffee. I think it’s that motivation.’	Skills (practice)<br>Beliefs about consequences<br>Reinforcement<br>Intention<br>Goals<br>Behavioural regulation	2,4
**Barriers to routine**			
**other health concerns**	(8) ‘I’ve not been very well as well; I’ve got diagnosed with kidney stones two weeks ago. The paramedics had to come; I was in so much	Environmental context and resources<br>Emotion	1,8

	pain. Although I'm not using it as an excuse, when things are suddenly thrown at you like that, it sort of puts all your routine out really, of doing stuff and that.'			
**Memory**	(4) 'One of the things that was really hard though was actually remembering them properly because they gave me sheets of diagrams, but it was quite hard'	Environmental context and resources<br>Memory, attention and decision processes	2,4,7,8,10,<br>11,13	
**Other commitments (work)**	(10) 'And because there were times, I was rushing off to work and I'd come back physically tired and mentally tired, because I was doing sign language. And I was coming home so shattered, I just wasn't always doing the exercises.'<br><br>(12) 'I've done them in the mornings, I've done them, it's awkward for me with shifts. I've done them at various different times of the day. I wouldn't say I've got a set pattern where I get up in the morning and I do them and I think, I feel great through the day.'	Environmental context and resources	6,10,12,13	Y
**Other commitments (non-work)**	(4) 'If you've got too many obligations, like mother and grandchildren, that would stop you getting in a routine.'	Environmental context and resources	1,4	
**Time**	(4) Well, in terms of the amount of times, yes, because at first, I was doing them every day. And then, when I came back on about the fourth visit and he was adding, adding, I said, "Well, it takes quite a long time now, it's taking about half an hour to do them."<br>(12) they've not been strenuous what they've given me. You can fit it in with five or 10 minutes. He said, "Just do a few reps at a time," he hasn't given me drastic amounts. It's not like he said, I want you to do half an hour or an hour.	Environmental context and resources	2,4,10,12,13	Y

Table 3: Post-Treatment Factors

**Theme/**sub-theme	**Example of quotation (participant no; 'quotation')**	**Associated TDF Domains**	**Participant Numbers**	**Conflicting Belief (y/n)**	**Strong Belief (y/n)**
**Access to resources**					
Follow up/review with physiotherapist	(10) 'then at least you know you can go back if something happens, because she says to me, "If anything happens in between then come back."'	Knowledge<br>Skills<br>Environmental context and resources<br>Social influences	2,10,7,11,12		
Facility to maintain PA	(2) 'I think it's a niche and it seems to be, provision for people who, for older people to do arm stretches and things like that. Then there's a gym for people who are younger and physically active, but where you get access to those pieces of equipment and that sort of expertise, I don't know anywhere else apart from this, which is why it's a goldmine….If they had information like that, I would grab it with both hands.'	Knowledge<br>Reinforcement<br>Environmental context and resources<br>Social influences	1,2,4,5,6,10,11		Y

Ease of Access: (Convenience, cost, special equipment needed etc)	(9) 'It's been a little bit more than normal because I've got the bike. I've deliberately got it set up so that, if I'm in the mood, I can just get on the bike, and sit and watch TV. So, rather than just sitting and watching TV or whatever I'm doing, I can sit there and I'm exercising my hip.'	Reinforcement<br>Intentions<br>Environmental context and resources	2,9,10	
**Psychosocial support**				
Relatedness to peers	(2) 'There's a new swimming baths opening in Northfield and there's a gym. I watch people who go in and out to see if any of them look like me and so far, I haven't seen anybody look like me.'<br><br>(10) 'A group, where people with knee pain, for instance, get invited to a session to do something. People with back pain and it would be sessions aimed at their specific problem, to help as a group. And also, that you'd be able	Environmental contexts and resources<br>Social influences<br>Social identity<br>Beliefs about capabilities<br>Optimism	1,2,4,5,6,10,13	Y

	to sit and chat about what you're having, that you're not the only one that's getting that pain or, "I'm getting the pain and I do this and that helps me." Like for instance, with my gel. If I say to people, "I use that." "Oh, I'll give that a go." You might be able to help people out that way.'			
Fear of judgement by others	(8) 'Because nobody really is interested in someone who's got chronic pain because it's quite boring isn't it? It's not like you've broken your leg and people go, "Oh, how long have you got that cast on for?" You know, in your circle of friends, I mean. Nobody wants to hear that your knee hurts, do they?'<br><br>(2) 'I don't want to feel stupid or embarrassed when I exercise.'	Social identity<br>Environmental context and resources<br>Social influence<br>Beliefs about Capabilities<br>Optimism<br>Emotion	2,7,8,10	Y
Fear of decreased support post-treatment	(2) 'I suppose I've got an image in my mind that I'd been given all this	Social identity<br>Environmental context and resources	2,8,10	Y

	amazing care and suddenly I'm going to go out, you can't see what I do there. It's outer darkness really and having to go and it's not having that support and vital information, I think.'	Social influence<br>Optimism<br>Emotion			
Family support	(10) 'It's usually not too long because my husband doesn't like walking, I think it's probably about half an hour'	Environmental context and resources<br>Social influence	8,10,11	Y	
**Physical Capability**					
Perceived physical Capability	(4) 'I know I'll hold them up and what happens is you struggle up the hill towards them, where they've been waiting for you for five minutes. As soon as you get there, they've had their rest, so off they go again, and you have to carry on up the hill. Yeah, so that does stop me doing things.'	Beliefs about Capabilities<br>Beliefs about Consequences<br>Environmental context and resources<br>Social influence<br>Emotion	4,7,8		
Fear of going out.	(9) 'Most of the time I don't need a stick, but the thing is, when…I can have times when this hip is so painful and my leg	Beliefs about Capabilities<br>Beliefs about Consequences<br>Environmental Context and resources	2,7,9	Y	Y

	feels as if it's going to support me. The stick isn't to help me walk properly. The stick is to give me confidence that, if I get that feeling in my leg, that I am not going to fall over.'				
Intrinsic Physical factors (age, weight)	(6) 'My weight is stopping me, because since I have stopped, the weight has piled on. I wasn't nowhere near as big then as I was now. Also, I gave up smoking and that's when the weight started to pile on as well.'	Beliefs about Capabilities<br>Beliefs about Consequences	2,5,6,7,8		
External physical factors (hills, uneven ground, stairs, heavy equipment)	(6) 'Whilst I'm okayish walking on the flat, one guy who organises our walks always says' "No, no, there's no hill, it's fine." And I say, "Well, yes, that's fine for you because your legs don't hurt, you don't even know that it's a hill." To me a hill is like that, to him a hill's like that.'	Beliefs about Capabilities<br>Beliefs about Consequences<br>Environmental Context and resources	1,4,6,8		Y
Weather (e.g. amount of light, cold)	(6) 'I think if I try and use my car less because I do only work down the road. The only reason I	Beliefs about Capabilities<br>Environmental context and resources	2,5,6,11		

do use my car in the mornings is because it's so dark and I don't really like walking down there.'

(5) 'The only thing I'll do out in the garden is I'll feed the birds, fill up the bird bath. But if it's too cold I'm back in. I don't like doing it when it's cold, it's worse now.'
